# Deep Sequencing MicroRNAs from Extracellular Membrane Vesicles Revealed the Association of the Vesicle Cargo with Cellular Origin

**DOI:** 10.3390/ijms21031141

**Published:** 2020-02-08

**Authors:** Uyen Thi Trang Than, Dominic Guanzon, James A Broadbent, Tony J Parker, David I Leavesley

**Affiliations:** 1 Vinmec Research Institute of Stem Cell and Gene Technology, Vinmec Health Care System, Ha Noi 10000, Vietnam; v.uyenttt@vinmec.com; 2 Tissue Repair and Translational Physiology Program, Institute of Health and Biomedical Innovation, Queensland University of Technology, Kelvin Grove, QLD 4059, Australia; dominic.guanzon@gmail.com (D.G.); a.parker@qut.edu.au (T.J.P.); 3 School of Biomedical Science, Faculty of Health, Queensland University of Technology, Kelvin Grove, QLD 4059, Australia; james.broadbent@gmail.com; 4 Skin Research Institute of Singapore, Agency for Science, Technology and Research, 8A Biomedical Grove, Singapore 138648, Singapore

**Keywords:** microRNA, extracellular membrane vesicle, keratinocytes, wound healing

## Abstract

Extracellular membrane vesicles (EVs) have emerged as potential candidates for diagnostics and therapeutics. We have previously reported that keratinocytes release three types of EVs into the extracellular environment. Importantly, those EVs contain a large number of microRNAs (miRNAs) as cargo. In this study, we examined the expression level of keratinocyte-derived EV miRNAs, their target genes and potential functions. Next generation sequencing results showed that over one hundred miRNAs in each EV subtype exhibited greater than 100 reads per million (RPM), indicating a relatively high abundance. Analysis of the miRNAs with the highest abundance revealed associations with different keratinocyte cell sources. For instance, hsa-miR-205 was associated with the HaCaT cells whereas hsa-miR-21, hsa-miR-203, hsa-miR-22 and hsa-miR-143 were associated with human primary dermal keratinocytes (PKCs). Additionally, functional annotation analysis of genes regulated by those miRNAs, especially with regard to biological processes, also revealed cell-type-specific associations with either HaCaTs or PKCs. Indeed, EV functional effects were related to their parental cellular origin; specifically, PKC-derived EVs influenced fibroblast migration whereas HaCaT-derived EVs did not. In addition, the data in this current study indicates that keratinocyte-derived EVs and/or their cargoes have potential applications for wound healing.

## 1. Introduction

Keratinocytes are the predominant cell type in skin, which has the primary role to protect the body from environmental damage. Keratinocytes, including the HaCaT cell line and Primary Keratinocyte Cells (PKCs), are known to release EVs into the extracellular environment [1,2,3]. EVs are nanoscale particles ranging from 30 nm to 5000 nm in diameter that are released from various cell types [4]. EVs are classified into three categories, including apoptotic bodies (APs, 1–5 µm), shedding microvesicles (MVs, 100–1000 nm) and exosomes (EXs, 30–150 nm) [5]. These three EV types are products of different biological processes; for instance, APs are products of apoptosis; MVs, also known as “ectosomes”, are shed directly from the cellular membrane; and EXs are products of endocytosis, and re-release into the extracellular environment by exocytosis [5]. Importantly, evidence suggests that EVs have roles in many biological processes, such as tissue repair, embryonic development, pregnancy, immune response, cancer development and various others [6]. For example, EVs have been implicated in cutaneous wound healing processes through their influence on coagulation, angiogenesis, cell migration, cell proliferation and ECM production [7]. As such, understanding the molecular cargo of EVs released from keratinocytes is pivotal to enable further investigation of EV function.

Genetic molecules, including microRNAs (miRNAs), messenger RNAs (mRNAs) and DNA, have been discovered in EVs [7,8] According to Vesiclepedia, at least 10,520 EV miRNA molecules have been detected and identified (http://microvesicles.org/, v3.1, released 15/8/2018). miRNAs are small non-coding RNAs, 22–25 nucleotides in length and which function to regulate the translation of mRNAs by binding to their 3′ untranslated region [9]. The key concept in support of EV miRNAs is that these genetic molecules are delivered by EVs from parental cells to recipient cells [8,10] where they might suppress or regulate gene expression post-transcriptionally, leading to changes in recipient cell behaviour. For example, mesenchymal stem cell-derived exosomes were found to transfer miRNA-125a to endothelial cells, which suppressed the production of angiogenic inhibitor delta-like 4 and thereby promoted the formation of endothelial tip cells and modulation of angiogenesis [10].

Despite there having been several investigations of EVs released from keratinocytes [1,2,3,11], there are only two publications describing EV miRNAs derived from keratinocytes [3,12]. Thus, there remains a lack of knowledge about keratinocyte-derived EV miRNAs and their involvement in skin physiology and pathology. In this study we present data obtained from the analysis of the miRNA profiles from the three EV populations isolated from HaCaT and PKCs. Our analysis revealed that the EV miRNA component is parental-cell specific and suggested that the EVs from different parental cells might elicit different functions on recipient cells. For example, we report that keratinocyte-derived EVs promoted the migration of human primary dermal fibroblasts in a cell-type-dependent manner.

## 2. Results

### 2.1. HaCaT- and PKC-Derived EVs Exhibit Common Highly Abundant miRNAs

The utilisation of deep-sequencing technology resulted in the identification of hundreds of EV-derived miRNAs. While the determination of whether all confer bioactivity is an important question, one previous study suggested that only highly abundant miRNAs have sufficient competitiveness for the miRNA–mRNA interaction to significantly suppress their targets [13]. Thus, in an attempt to better understand which populations of EV miRNAs might elicit substantial bioactivity, an analysis of the miRNA abundance (represented by total read count number) within each EV population was conducted. There were 181, 186 and 189 miRNAs with greater than 100 Reads Per Million (RPM) and 78, 78 and 69 miRNAs with greater than 1000 RPM from HaCaT-derived APs, MVs and EXs, respectively (Table 1). With regard to PKC-derived EVs, there were 210, 214 and 210 miRNAs that exhibited greater than 100 RPM and 82, 81 and 79 miRNAs having greater than 1000 RPM in APs, MVs and EXs, respectively (Table 1). Thus, it may be hypothesised that these highly abundant miRNAs confer detectable bioactivity and elicit functional responses in other cells and tissues.

These data also showed that the majority of the five most abundant miRNAs were common to all EV released from both cell types, including hsa-miR-21, hsa-miR-22 and hsa-miR-27b (Table 2). Further the most abundant miRNAs that exhibited specificity with cellular origin, included hsa-miR-205 in HaCaT-derived EVs (*p <* 0.001) and hsa-miR-203 from PKC-derived EVs (*p <* 0.001). In addition, hsa-miR-181 was in the top five most abundant miRNA in HaCaT-derived APs and MVs, whereas hsa-miR-92 was in the top five most abundant miRNA in HaCaT-derived EXs (Table 2). With regard to PKC-derived EVs, hsa-miR-143 was in the top five most abundant in APs and MVs but did not rank in the PKC-derived EXs, whereas hsa-miR-205 was in the top five most abundant in the PKC-derived EX population but not in the top five most abundant miRNAs of APs and MVs released by PKCs (Table 2). Taken together these data indicate that the three EV populations from both keratinocyte cell types are largely enriched with the same miRNAs, which potentially suggest that the majority of miRNA mediated bioactivities of HaCaT- and PKC-derived EVs might also be the same.

### 2.2. Specific EV miRNAs Are Correlated with Cellular Origin

**Figure 1:**
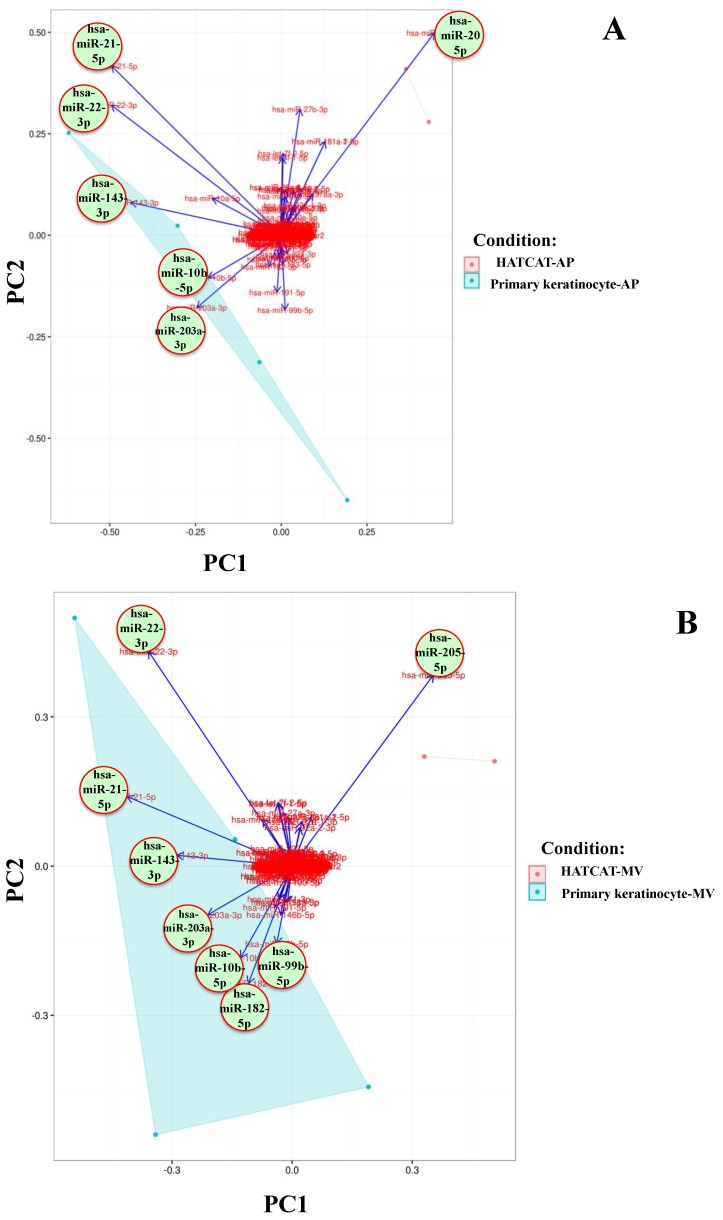

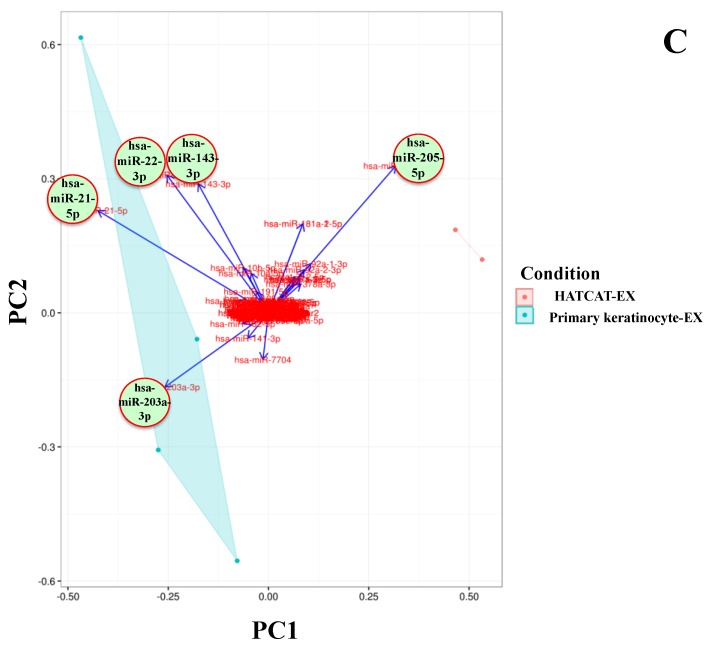
miRNA-205 is more associated with HaCaT cells and miRNA-21, miRNA-203, miRNA-22 and miRNA-143 are more associated with PKCs. Identified miRNAs from: (**A**) APs; (**B**) MVs; and (**C**) EXs. Biological replicates for HaCaT (n = 2) and PKCs (n = 4).

In order to determine whether the identified miRNAs explained the difference between the EVs of each parental cell line, the read count of identified miRNAs was analysed using principal component analysis (PCA). Loadings plots indicated that the majority of identified miRNAs did not substantially affect the difference between EV types from each parental cell type. hsa-miR-21, hsa-miR-203, hsa-miR-22 and hsa-miR-143 exhibited eigenvectors that were associated with PKCs in all three EV types while the hsa-miR-205 eigenvector was more strongly associated with HaCaT cells across all EV types (Figure 1). These miRNAs also exhibited the highest RPMs as reported in Table 2; thus, the PCA data was largely consistent with the RPM analysis suggesting that the relative abundance of these specific miRNAs is related to the cell type of origin.

Upon closer examination, while hsa-miR-205 was most associated with all three HaCaT-derived EV types (Figure 1A–C), hsa-miR-21, hsa-miR-22, hsa-miR-143, hsa-miR-203, hsa-miR-10b and hsa-miR-205 exhibited the greatest eigenvector values in the PKC-derived AP populations (Figure 1A). Similarly, the eigenvectors of hsa-miR-22, hsa-miR-21, hsa-miR-143, hsa-miR-203, hsa-miR-10b, hsa-miR-182 and hsa-miR-99b were most strongly associated with differentiation of the PKC-derived MV population (Figure 1B). Interestingly, only hsa-miR-21, hsa-miR-203, hsa-miR-22 and hsa-miR-143 exhibited eigenvectors that were more associated with PKC-derived EXs; indeed, only these four miRNAs were common to all PKC-derived EVs (Figure 1C). Thus, these EV miRNAs appeared to be the most responsible for differentiating between PKC-derived and HaCaT-derived EVs. As such they were subjected to bioinformatics analysis to determine their potential target genes and therefore their potential bioactivities.

### 2.3. Many Target Genes Regulated by miRNAs Associated with HaCaT and PKCs

**Figure 2:**
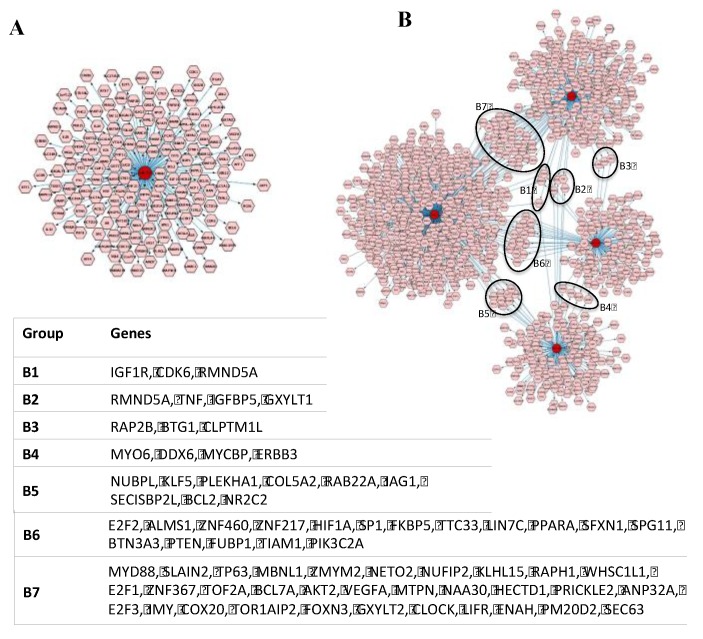
Network of target genes regulated by (**A**) hsa-miR-205 and (**B**) by a group of four miRNAs, including hsa-miR-21, hsa-miR-203, hsa-miR-22 and hsa-miR-143. Inset table indicates gene groupings regulated by at least (B1) three or (B2–B7) two miRNAs.

In order to investigate the potential difference in bioactivity of EVs derived from each parental cell type, EV miRNAs were analysed for their known target genes using miRTarbase20. The results indicated that hsa-miR-205 is known to regulate 168 target genes (which encoded for 173 proteins) (Figure 2A; Supplementary Table S1), while collectively the four miRNAs, hsa-miR-21, hsa-miR-203, hsa-miR-22 and hsa-miR-143, are known to regulate 1076 genes (encoding for 1098 proteins) (Figure 2B; Supplementary Table S2).

In order to organise these large sets of target genes into functionally related groups, they were further analysed using a Functional Classification Tool (DAVID Bioinformatics Resources v6.8). There were three functional groups classified from the target genes regulated by hsa-miR-205. The first group (enrichment score 2.72) is involved in protein kinase activity, cytoplasm, nucleus, membrane, metal, ATP and nucleotide binding. The second functional group (enrichment score 2.01) is involved in the zinc finger, nucleic acid/DNA/metal binding, repressor, activator, transcription and nucleus. Finally, the third group (enrichment score 1.08) is related to the plasma membrane, component of membrane, transmembrane, topological domain: cytoplasmic/extracellular, blood coagulation and haemostasis (Table 3). This suggests that HaCaT-derived vesicles are involved in those activities of binding, membrane, nucleus and haemostasis through hsa-miR-205.

Additionally, 36 functional groups were classified from the target genes regulated by the four miRNAs associated with PKCs (Table 4). The highest ranked group was related to cytoplasm, nucleus, protein binding and phosphoprotein (enrichment score 27.64). The next group was associated with transcription, nucleus, protein binding, DNA binding and phosphoprotein (enrichment group score 19.96). However, the most common biological functions that appeared in all 36 classified groups included those involved in the nucleus, membrane, cytoplasm, binding activity and zinc finger. Additionally, others such as phosphoprotein, signalling pathway, transcription, kinase activity, transmembrane, cytosol and transport are the second most common biological functions that appeared in all functional groups (Table 4). This information indicates that PCK-derived EV miRNAs may participate in regulating biological processes at the nucleus, cytoplasm and membrane; for example, activities of phosphorylation, kinase and binding.

### 2.4. Regulatory Roles of EVs

**Figure 3:**
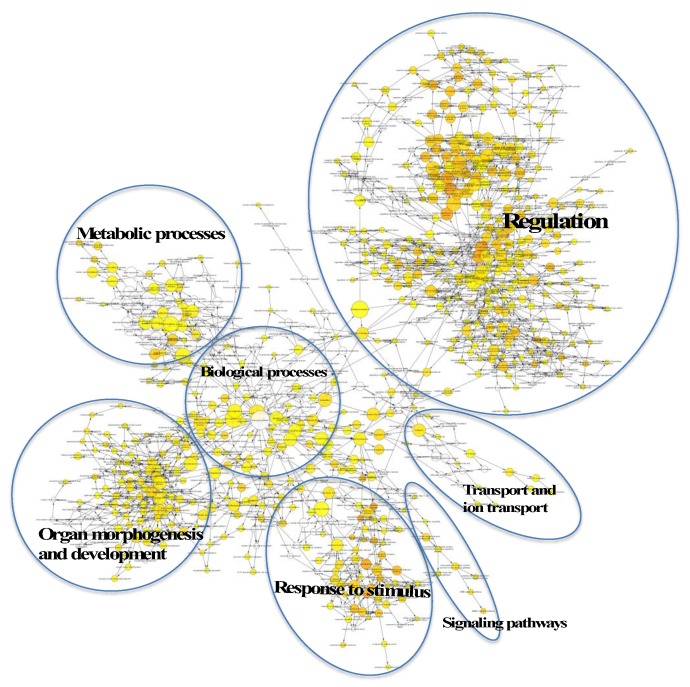
Visualisation of the biological processes (BPs) resulting from the analysis of target genes regulated by hsa-miR-205 associated with HaCaT-derived EVs using Cytoscape (Version 3.2.1).

**Figure 4:**
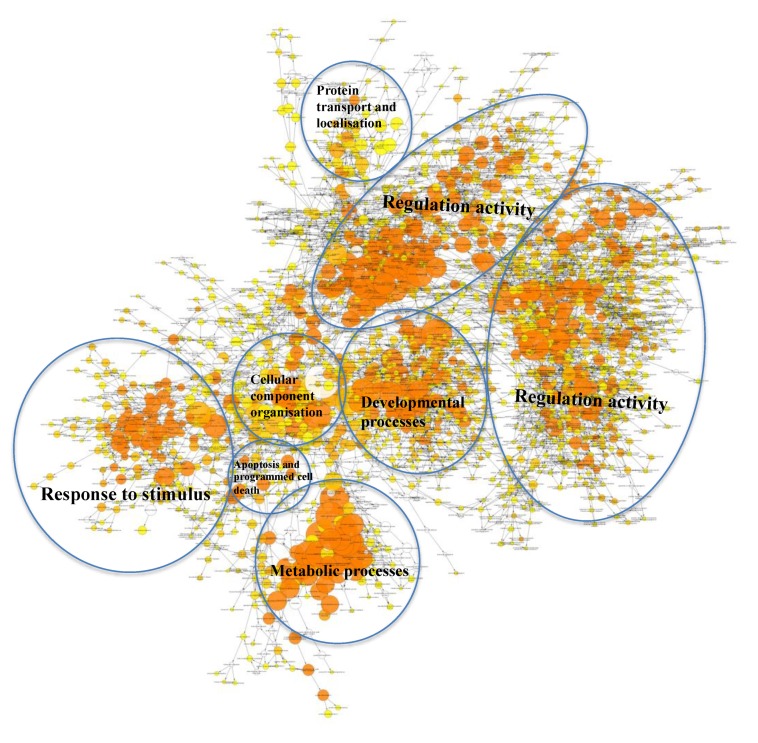
Visualisation of the BPs resulted from the analysis of target genes regulated by a group of four miRNAs (hsa-miR-21, hsa-miR-203, hsa-miR-22 and hsa-miR-143) associated with PKCs using Cytoscape (Version 3.2.1).

In order to determine the potential biological processes (BPs) regulated by the target genes of the EV miRNA that were associated with HaCaT and PKCs (from Figure 2), a gene ontology (GO) analysis was performed using Cytoscape BiNGO (v3.2.1). The BP terms associated with the target genes regulated by hsa-miR-205, and therefore EVs derived from HaCaT cells, were mostly related to regulation, organ morphogenesis and development, metabolic processes, response to stimulus, transport and signalling pathways (Figure 3). Regarding the target genes regulated by the four miRNAs, which are highly enriched within PKCs, the BP analysis revealed that they were associated with regulation activity; response to stimulus; developmental process; metabolic process; protein transport and localisation; cellular component organization and apoptosis; and programmed cell death (Figure 4).

**Figure 5:**
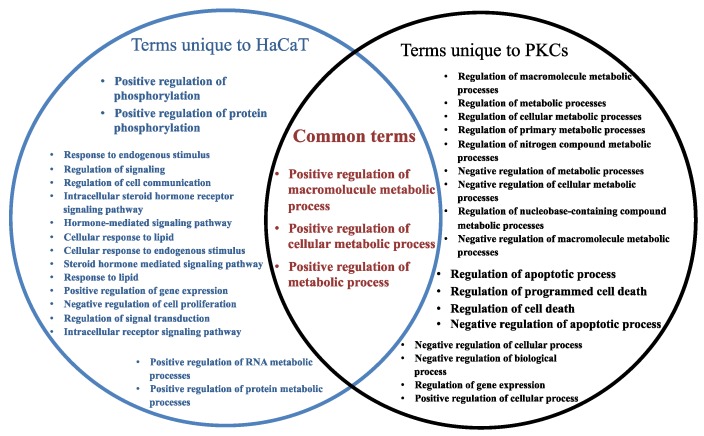
Common and unique terms associated with HaCaT- and PKC-derived EVs. The reported terms herein are from the 20 most statistically significant GO Terms. The red text indicates common terms associated with the target genes of miRNA from HaCaT- and PKC-derived EVs.

A report of the 20 most significantly over represented BP terms associated with the target genes of the highly abundant EV-derived miRNAs from HaCaT and PKCs revealed three common terms between the two cell types, including positive regulation of macromolecule metabolic process, positive regulation of cellular metabolic process and positive regulation of metabolic process. This indicates that these highly abundant EV miRNAs may impact recipient cell metabolic activity through regulation of the translation of their target genes. Interestingly, the unique terms overrepresented by the target genes of miRNA associated with HaCaT-derived EVs were linked to regulation of phosphorylation and steroid hormone pathways; in turn, the unique terms overrepresented by the target genes of miRNA associated with PKC-derived EVs were associated with metabolic processes, apoptosis and cell death (Figure 5,  Supplemental Table S3).

### 2.5. Target Genes Regulated by EV miRNAs Related to Cell Migration

In order to examine the association of keratinocyte-derived EVs with wound healing through fibroblast migration, we further analysed the obtained BP terms (from Figure 3 to Figure 4) to find any terms related to cell migration. The data revealed that that the target genes regulated by hsa-miR-205, which was associated with EVs derived from HaCaT cells, was enriched in six cell migration-related terms. In addition, the four miRNAs (hsa-miR-21, hsa-miR-203, hsa-miR-22 and hsa-miR-143) that were associated with PKC-derived EVs were enriched in 25 migration terms (Table 5). This suggests that EVs from both cell types may contribute to some extent in the regulation of cell migration. Importantly, the term “Regulation of fibroblast migration” was found to be enriched only in the PKC-derived EV miRNAs.

**Figure 6:**
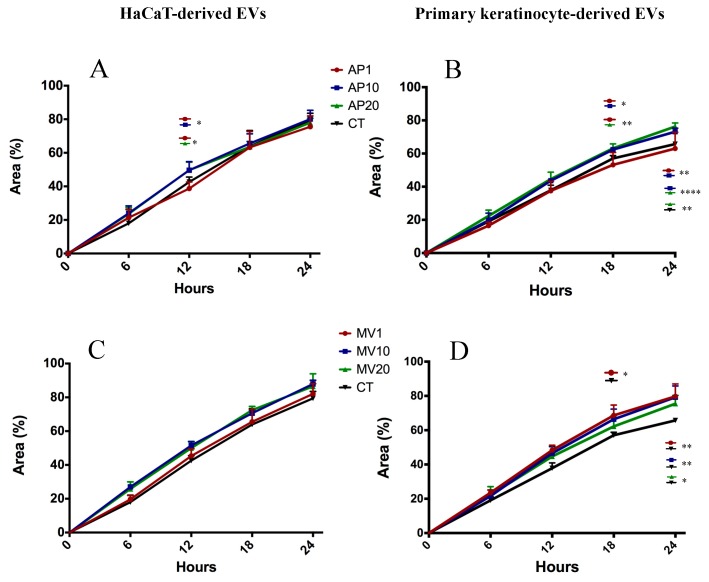

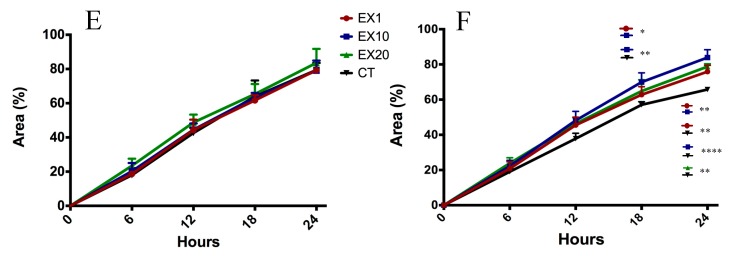
Primary dermal fibroblast migration is modulated by PKC-derived EVs but is not dose dependent. HaCaT- or PKC-derived (**A**,**B**) APs, (**C**,**D**) MVs or (**E**,**F**) EXs were added to scratch-wounded primary fibroblast cultures to a final total EV protein concentration of 1 µg, 10 µg or 20 µg/0.1 mL DM as indicated. The fibroblasts were incubated at 5% CO_2_ and 37 °C and allowed to migrate for 24 h with images captured at 6 hourly intervals. Image analysis was performed using ImageJ and data are presented as the mean percent area of wound coverage in µm^2^ ± SD, from at least 3 independent biological replicates. Two-way ANOVA and Tukey’s multiple comparison tests were used to evaluate statistical significance, which is denoted as * where *p <* 0.05; ** *p <* 0.01; and **** *p <* 0.0001. AP: Apoptotic bodies; MV: Microvesicles; EX: Exosomes; CT: Control.

We validated the influence of keratinocyte-derived EVs on human primary dermal fibroblast migration using a scratch wound assay created in a monolayer of dermal fibroblasts and subsequently treated with keratinocyte-derived EVs. We found that, in general, the migration of fibroblasts treated with HaCaT-derived EVs were indistinguishable to the untreated controls (Figure 6A,C,E). In contrast, observations of primary fibroblasts treated with PKC-derived EVs revealed that the fibroblasts generally migrated more in response to EV treatment and the extent of cell migration was dependent on EV types and doses (Figure 6B,D,F). This indicated that the capacity of EVs to enhance human dermal fibroblast migration depends on the EV cellular origin.

**Figure 7:**
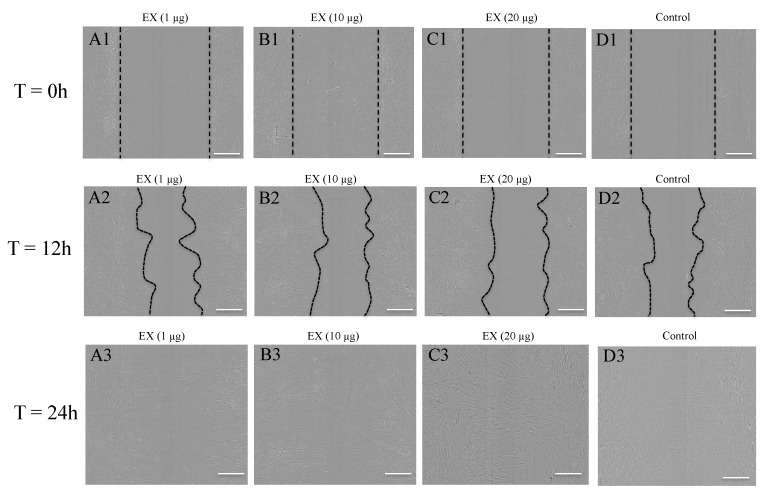
Primary keratinocyte-derived EXs facilitate migration of dermal fibroblasts in 2D culture. Isolated primary keratinocyte-derived EXs were added to scratch-wounded primary fibroblast cultures to a final total EV protein concentration of 1 µg, 10 µg or 20 µg/0.1mL depleted media. Fibroblasts cultured within depleted media only served as controls. The wounded fibroblast cultures were incubated at 5% CO_2_ and 37 °C and allowed to migrate for 24 h in the presence or absence of primary keratinocyte-derived EXs with images captured at 6 hourly intervals. Representative images were extracted from the captured IncuCyte^TM^ images at (**A1**,**B1**,**C1**,**D1**) T = 0 h; (**A2**,**B2**,**C2**,**D2**) T = 12 h; and (**A3**,**B3**,**C3**,**D3**) T = 24 h. Images were captured using a 4X objective. The scale bar = 300 µm and the dotted lines represent the wound edge.

Of interest, all three EV populations derived from PKCs induced significant fibroblast migration compared to the controls (Figure 6B,D,F and Figure 7). Specifically, PKC-derived APs did not enhance fibroblast migration at doses of 1 µg/0.1 mL or 10 µg/0.1 mL, but did significantly elevate cell migration after 24 h exposure to a dose of 20 µg/0.1 mL compared to the untreated control group (*p <* 0.01) (Figure 6B). The fibroblasts migrated further after 18 h when treated with 1 µg/0.1 mL of PKC-derived MVs (*p <* 0.01), or after the 24 h when treated with 1 µg/0.1 mL (*p <* 0.001) or 20 µg/0.1 mL (*p <* 0.05) of PKC-derived MVs (Figure 6D). Enhanced cell migration was also observed in fibroblast cultures after 24 h treatment with 1 µg/0.1 mL (*p <* 0.01), 10 µg/0.1 mL (*p <* 0.0001) and 20 µg/0.1 mL of PKC-derived EXs (*p <* 0.001) (Figure 6F, 18 h & 24 h). However, the response of human primary dermal fibroblasts to individual EV populations was not clear when a comparison of cell migration was made between APs, MVs and EXs released from the same donor cell source and at equivalent concentrations (data not shown).

Taken together, these data indicate that while PKC-derived EVs generally enhanced the migration response of fibroblast cells, the dose responsiveness was relatively inconsistent and therefore did not appear to be dose dependent. Moreover, the extent of the migration response of fibroblasts to EVs is dependent on the EV parental cell source and the duration of the exposure to the EVs.

## 3. Discussions

Over the past few years, several studies have described the expression of miRNAs in EVs [14,15], and recently EV miRNAs have evidently modified functional measures in recipient cells [10]. Our previous study characterised three EV populations released from HaCaT and PKCs and reported that these EVs carry many miRNA species [3,16]. In this current study, we show miRNAs that exhibited high abundances and that were identified in the three EV populations, as well as that these specific EV miRNAs were associated with their parental cellular origin. Of these, approximately 200 EV miRNAs exhibited greater than 100 RPM and 100 miRNAs exhibited greater than 1000 RPM. Despite the large number of abundant miRNAs identified in this current study, how many, and/or which of these are active and regulate target mRNAs, remain an important question. As reported previously, only highly expressed miRNAs seem able to significantly regulate their targets [13,17,18]. For instance, Brown et al. demonstrated that only miRNAs that have greater than 100 copies/pg of the short RNA fraction significantly suppressed their targets [17]. Similarly, Mullokandov et al. showed that 80% of miRNAs that have greater than 100 RPM have suppressive activity [13]. In addition, and to further complicate matters, not all highly expressed miRNAs suppress their targets; for example, miRNA-223 was similarly highly expressed in U937 monocyte cells, 293T kidney cells and HuH7 liver cells, but only suppressed its target mRNA in monocyte cells and not in the other cell types [17]. Therefore, the specific suppressive activity, or otherwise, of the miRNAs described in this study is promising but need to be validated.

With regard to the miRNA populations identified in all three keratinocyte-derived EV populations, our previous research indicated that there were 242 unique miRNAs associated with HaCaT-derived EVs and 56 unique miRNAs associated with PKC-derived EVs [3] Importantly, among the most abundant miRNAs, hsa-miR-205 was highly associated with HaCaT whereas the four miRNAs, hsa-miR-21, hsa-miR-203, hsa-miR-22 and hsa-miR-143, were highly associated with PKCs. Several of these miRNAs have been previously reported to be involved in keratinocyte and skin biology [19,20,21,22]. The miRNAs (miRNA-205, miRNA-21 and miRNA-203) have been previously found to induce keratinocyte migration, proliferation and differentiation through regulation of various gene targets [19,21,22,23]. For example, hsa-miR-205 directly silences SH2-domain containing inositol 5-phospatase 2 (SHIP2), resulting in an increase in cell migration [21]; hsa-miR-21 targets the inhibitory Smad and Smad 7 to promote collagen inhibition [24], or targets phosphatase and tensin homolog (PTEN) [25], causing skin fibrosis by fibroblasts [26]; and hsa-miR-203 regulates p63, Ras-related nuclear protein-Ras association (RalGDS/AF-6) and pleckstrin homology domains 1, as well as LIM and SH3 domain protein 1 (LASP1), which are responsible for both keratinocyte proliferation and migration during wound re-epithelialization [23]. Furthermore, miRNA-205 is highly expressed in skin cancer (cutaneous squamous cell carcinoma) [27], and miRNA-21 is highly expressed in injured skin and enhances re-epithelialisation [20,22]. These observations may indicate that the EV miRNAs found to originate from both HaCaT cells and PKCs in this current study all have a role in wound healing and skin biology.

These miRNAs have been demonstrated to be involved in many biological events despite the fact that very little practical evidence regarding the association of EV miRNAs and biological events is available; thus, bioinformatic analysis of the genes regulated by EV miRNAs can potentially provide insight as to the relative contribution of EVs and EV miRNAs to various biological activities. For instance, preliminary bioinformatic analysis of genes targeted by miRNAs detected in LIM1863 colon cancer cell line-derived EVs resulted in various enrichment of GO terms, such as extracellular matrix, membrane and cancer progression [14]. Furthermore, important pathways, such as the p53 signalling pathway, TGF-beta signalling pathway, MAPK signalling pathway, cell cycle, among others, have been reported previously as having an association with miRNA-mediated regulation [14,28,29]. In this present study, the BP GO terms enriched by the EV miRNA target genes are associated with the regulation activity, metabolic process, biological process, response to stimulus, organ morphogenesis and development, transport and signalling pathway. However, there are also distinct BP GO terms associated with EV parental cell origin. These disparities may arise from the specific differences in the physiological conditions between the HaCaT cell line, which resemble transformed keratinocytes, and primary keratinocyte cells, that while more biologically relevant exhibit heterogeneity between donors [30,31]. Both the experimental and bioinformatic data indicated that fibroblast migration may be regulated by PKC-derived EVs but not by HaCaT-derived EVs, which demonstrates the association of biological processes with EV cellular origin. It is noted that the release of EV and selective sorting of molecular cargo, including miRNAs, into EVs upon stimulation in an unrelated cell type supports the wider role of EVs in human physiology [32,33]. Although bioinformatics information in this current study may serve to indicate the potential connections between EV miRNAs and functional consequences, it is important that experimental corroboration is obtained to have confidence that EV miRNA regulate target genes and biological functions.

Together, the EV miRNA data presented herein provide valuable insights into the EV miRNA composition and point to the potential activity of EV miRNAs in vivo. Data arising from this study revealed differentially abundant EV miRNA populations associated with particular cellular types. Importantly, the target genes of the most abundant EV miRNAs differ from their classified function and encode for some important proteins associated with many biological activities, including dermal–epidermal wound healing and fibroblast migration. While the keratinocyte-derived EVs utilised in this study were not produced from a robust wound healing model, the data does still suggest that some EV miRNAs do have a role during cutaneous wound healing.

## 4. Materials and Methods

### 4.1. Cell Culture

Ethical approval for research detailed herein was obtained from Queensland University of Technology (QUT); Pacific Day Surgery/Brisbane Private Hospital (approval # 1300000063/QUT); Princess Alexandra Hospital (approval # HREC/06/QPAH/91); and Uniting Health Care’s St. Andrews Hospital and Wesley Hospital (approval # 2003/46).

Epidermal primary keratinocytes (PKCs) were freshly isolated from donor skin (surgical discard) and propagated on i3T3 feeder cells (1 × 10^6^ cells/T75 flask) using the method described by Rheinwald and Green [34]: DMEM | Ham’s F12 (3:1), supplemented with 10% foetal calf serum, 2 mM L-glutamine, 1% v/v penicillin–streptomycin, 180 μM adenine, 0.5 μM insulin, 0.05 μM cholera toxin, 0.01% v/v non-essential amino acids solutions, 2.5 μg transferrin, 0.1 μM triiodothyronine, 0.16 μg hydrocortisone and 0.05 ng human recombinant endothelial growth factor [34].

HaCaT cells were purchased from CLS Cell Lines Service GmbH (Eppenheim, Germany) [35]. HaCaT cells seeded at 1−2 × 106 cells/T75 flask were propagated in DMEM supplemented with 10% FCS, 1% pen/strep and 1% glutamine).

All cultures for both PKCs and HaCaT were maintained at 37 °C in a 5% CO_2_/95% air atmosphere, re-fed every 2 days, and sub-cultured when cells reached 80% confluency.

### 4.2. EV Production and Isolation

EV production and isolation were reported in our previous publication [3]. Briefly, PKCs and HaCaT were cultured to 80% confluence; the expired media and i3T3 cells were removed and the remaining PKC cultures washed prior to incubation for 48 h with serum-free media for EV production. The EV-enriched media released by HaCaT and PKC cultures (conditioned media-CM) were collected and centrifuged at 300× *g* for 10 min to remove cell debris prior to proceeding with EV isolation [36].

EV enriched CM were gravity filtered through a 5 µm Durapore^®^ membrane filter (Millipore) then centrifuged at 3000× *g* for 40 min prior to collection and resuspension of the of the AP pellets in PBS, and re-centrifugation at 3500× *g* for 1 h. The resulting pelleted material was designated as clean “AP-harvest”. Supernatants from the AP-harvest were gravity filtered through a 1.2 µm Hydrophilic Nylon membrane filter (Millipore) prior to centrifugation of the filtrates at 16,500× *g* for 1 h at 4 °C (Rotor SW70, Beckman Coulter, Indianapolis, USA). The resulting “MV-harvest” pellets were resuspended in PBS and centrifuged at 16,500× *g* for 1 h at 4 °C prior to gravity filtration of resulting supernatants through a 0.1 µm Durapore^®^ membrane filter (Millipore). The filtrate was then centrifuged at 100,000× *g* for 1.5 h at 4 °C to yield “EX-harvest” pellets. The EX pellets were resuspended in PBS and centrifuged at 100,000× *g* for 1.5 h at 4 °C for clean EXs. Clean AP, MV and EX harvest materials were resuspended in PBS (approximately 30 µL) and stored at −20 °C for up to 4 weeks, or at −80 °C for longer-term storage.

### 4.3. Total RNA Extraction and miRNA Sequencing

The total RNA extraction using Trizol^TM^ method and small RNA sequencing using Illumina^®^ Next Seq500 were reported in our previous publication [3]. Briefly, total RNAs were extracted from vesicle pellets using the Trizol^TM^ method prior to being subjected to an Illumina^®^TruSeq^®^Small RNA Library Prep Kit as per the manufacturer’s instructions. The RNAs were ligated to adapters prior to reverse transcription to cDNAs and amplified by PCR. The amplified PCR products from this stage were referred to as the small RNA library, which was subsequently purified by gel electrophoresis. The bands containing miRNAs between 145 bp and 160 bp were excised and eluted in 200 µL pure water. The resulting cDNA library was diluted to 2 nM using a solution of Tris-HCl 10 nM, pH 8.5 and 0.1% Tween 20 prior to loading onto an Illumina chip and sequencing using an Illumina^®^ Next Seq500.

### 4.4. miRNA Identification and Statistics

The raw data generated from the Illumina^®^ Next Seq500 were exported into a FASTQ file. Index and adaptor sequences were then removed using the TagCleaner program (http://tagcleaner.sourceforge.net/index.html, version 0.16) and trimmed to 28 nucleotides using the FASTX-Toolkit program (http://hannonlab.cshl.edu/fastx_toolkit/index.html, version 0.0.13) prior to submission of the cleaned nucleotide data to the miRDeep2 software (version 1.10.1) for subsequent analysis. The sequences were aligned to the human genome (hg19, downloaded from http://bowtie-bio.sourceforge.net/index.shtml) using the mapper and quantifier modules in miRDeep2 to identify and quantified miRNAs.

The identified miRNAs and their raw counts were further analysed using a DESeq2 software and R statistical environment (R version 3.2.2, last update 14/8/2015) for filtering, normalisation and PCA analysis to test the correlation of identified miRNAs between EVs and their parental cell source.

### 4.5. Analysis of miRNA Target Genes

The miRNAs of interest were searched for its miRbase ID using the miRBase website (www.mirbase.org). These were subsequently analysed using Cytoscape (version 3.2.1) to create an interaction network of the identified miRNAs and their target genes. The target database (version 6.0) was downloaded from the miRTarBase website (accessed 5/1/2016, http://mirtarbase.mbc.nctu.edu.tw). miRTarbase is an experimentally validated miRNA-target interaction database that was updated recently (released 15/9/2015 for version 6). The data were exported as an image of the target gene-miRNA interaction network and as an excel file of target genes.

A target gene list was then classified into similar functional groups using the DAVID Bioinformatics Resources version 6.8 (https://david-d.ncifcrf.gov/) based on the whole Homo sapiens genome background [37,38]. The Functional Classification Tool was used with Kapa similarity term overlap set to 4 and Kapa similarity threshold set to 0.35, classification parameters were set to initial group membership at 4; final group membership at 4; multiple linkage threshold set to 0.5; and the classification stringer was set to medium.

The target gene list was also analysed for Biological Processes (BPs) and network visualisation using Cytoscape BiNGO (version 3.2.1). The Gene Ontology (GO) database was download from the official website of Gene Ontology (go-basic.obo, http://geneontology.org/page/download-ontology, downloaded 28/3/2017). The GO annotation database was downloaded from the Gene Ontology website (http://geneontology.org/page/download-annotations, version released 13/3/2017, downloaded 28/3/2017; go_basic.obg, goa_human.gaf (annotation), which contain 19,318 annotated products). The Benjamini and Hochberg False Discovery Rate was used for correction and the *p*-value cut off was at 0.05.

### 4.6. Scratch-Wound Assay

Primary human dermal fibroblasts isolated from individual human donors were harvested as described above and propagated until a sufficient number of cells (passage 4) were available to seed 96 well ImageLock plates (Essen BioScience, Michigan, USA). The fibroblast culture media used for the scratch assay cultures were comprised of similar components to normal fibroblast culture media (NM), which included DMEM, 10% FCS, 1% Pen/Strep and 1% L-glutamine. Since FCS may contain endogenous EVs that might influence cell function, depleted media (DM) was prepared by subjecting FCS to centrifugation at 120,000× *g* for 24 h at 4 °C before addition of the collected supernatant to culture media [39]. Cells were cultured to 100% confluence and the treated with 10 µg Mitomycin C (Sigma Aldrich) in order to inhibit further cell proliferation. Scratch wounds were then created simultaneously in each well using a WoundMaker™ device (Essen Bioscience). Closure of the “wounded” monolayer of primary human dermal fibroblasts, in the presence (and absence) of 1 µg, 10 µg or 20 µg EV protein per well (0.1 mL cell culture media), was monitored using an IncuCyte™ Zoom instrument (Essen Bioscience). CSV data was imported into Excel (Microsoft) and Wound Relative Density used to calculate wound closure parameters throughout the experiment. Image analysis was performed using ImageJ and data were presented as the mean percent area of wound coverage in µm^2^ ± SD, from at least 3 independent biological replicates. Two-way ANOVA and Tukey’s multiple comparison tests were used to evaluate statistical significance.

## Figures and Tables

**Table 1:** Number of highly abundant extracellular membrane vesicle (EV) miRNAs with reads per million (RPM) greater than 100 and greater than 1000.

Parental Cell Types		AP	MV	EX
**HaCaT**	>100 RPM	181	186	189
	>1000 RPM	78	78	69
**Primary keratinocytes**	>100 RPM	210	214	210
	>1000 RPM	82	81	79

**Table 2:** The relative abundance levels of the five most abundant miRNAs from HaCaT and primary keratinocyte-derived EVs.

**HaCaT**					
**AP**		**MV**		**EX**	
**miRNA Name**	**MN Counts ± SD**	**miRNA Name**	**MN Counts ± SD**	**miRNA Name**	**MN Counts ± SD**
hsa-miR-205-5p	106,506.6 ± 8267.9 ****	hsa-miR-205-5p	123,329.2 ± 44,355.9 ***	hsa-miR-205-5p	100,832.8 ± 16,630.3 *
hsa-miR-22-3p	92,576.7 ± 16,680 ****	hsa-miR-27b-3p	60,380.3 ± 11,723.7 **	hsa-miR-22-3p	77,505.6 ± 14,611.1 *
hsa-miR-27b-3p	57,661.6 ± 7889.6 ****	hsa-miR-22-3p	59,791.6 ± 4818.9 **	hsa-miR-27b-3p	51,490.9 ± 237.7 ***
hsa-miR-21-5p	49,946.5 ± 6634.3 *****	hsa-miR-21-5p	45,496.7 ± 3357 ***	hsa-miR-21-5p	49,943.9 ± 4831.5 ***
hsa-miR-181a-1-5p	48,805.9 ± 1721.1 *****	hsa-miR-181a-1-5p	45,541.5 ± 3393.9 ***	hsa-miR-92a-1-3p	37,858.4 ± 523.1 ****
**Primary Keratinocytes**					
**AP**		**MV**		**EX**	
**miRNA Name**	**MN Counts ± SD**	**miRNA Name**	**MN Counts ± SD**	**miRNA Name**	**MN Counts ± SD**
hsa-miR-22-3p	139,339.8 ± 43,597.7 ***	hsa-miR-22-3p	93,878.6 ± 29,984.7 ***	hsa-miR-22-3p	111,055.9 ± 9430.8 ******
hsa-miR-21-5p	81,954.5 ± 31,594.6	hsa-miR-21-5p	86,901.3 ± 21,422.5	hsa-miR-21-5p	110,112.7 ± 15,649.5
hsa-miR-143-3p	42,915.2 ± 24,838.4 **	hsa-miR-143-3p	38,016.7 ± 17,012.7 **	hsa-miR-27b-3p	52,399.1 ± 8430.5 ****
hsa-miR-203a-3p	40,830.8 ± 7053.9 **	hsa-miR-27b-3p	45,115.2 ± 3168.5 *	hsa-miR-203a-3p	49,640.4 ± 10,579.5 ****
hsa-miR-27b-3p	40,707.7 ± 12,724.9 **	hsa-miR-203a-3p	34,067.1 ± 13,381.3 **	hsa-miR-205-5p	44,981.6 ± 10,617.4 *****

MN counts: Mean of normalised read counts from biological repeats using the RPM method. RPM: Read Per Million. SD: Standard Deviation. Statistical significance was determined by ANOVA and Post-hoc Tukey HSD tests, and is indicated by * where *p <* 0.05; ** where *p <* 0.01; *** where *p <* 0.001; **** where *p <* 0.0001; ***** where *p <* 0.00001; ****** where *p <* 0.000001.

**Table 3:** Functional classification of genes regulated by hsa-miR-205.

Group (Enrichment Score)	Genes	Biological Function
Group 1 (2.72)	MAP3K9, NEK9, ICK, STK38L	Protein kinase, protein phosphorylation, binding, nucleus, cytosol, cytoplasm, membrane
Group 2 (2.01)	BCL6, ZEB1, ZRB2, YY1, PLAGL2	Zinc finger, zinc, repressor, activator, transcription, binding, nucleus
Group 3 (1.08)	SLC38A1, TMEM123, PAPPA-AS1, F2RL2, ENPP4, MMD, LYMSMD3, SLC5A12, SHISA6, NIPA2, TM9SF2, LRRTM4, SLC39A14, TMEM201, SMIM13, TMEM239, SERINC3, PRRG4, MANMI	Plasma membrane, transmembrane, topological domain: cytoplasmic, topological domain: extracellular, blood coagulation and haemostasis

**Table 4:** Functional classification of genes regulated by a group of four miRNAs, including hsa-miR-21, hsa-miR-203, hsa-miR-22 and hsa-miR-143, which are highly correlated with PKCs.

Group (Enrichment Score)	Genes	Biological Function
Group 1 (27.64)	PLEKHA2, NUDCD1, ELP5, FILIP1L, NUFIP2, SERBP1, SSFA2, DAZAP2, HYPK, MYCBP, TSC22D4	Cytoplasm, nucleus, protein binding, phosphoprotein
Group 2 (19.96)	ZBTB44, FAM208A, FOXK2, VGLL4, ZBTB8A, MAFK, MGA, LRRFIP1, MED9, NFAT5, ZNF654, ARID3B, MYCBP, HEXIM1, FOXN3, TAF1D, ATF7IP, DNTTIP2, CSRNP2, MIER3, NFYB, NFYA, ANP32A, FUBP1, ZNF646, TRAPPC2, PCGF6, ZNF367, NFIC, LCORL, TCEAL1, ELP5, HOMEZ, NFIA, BTF3, TSC22D4, SERTAD3, FOXN2, PURB, MACC1, PURA, FOXK1, HMGB3, DMTF1, COMMD2, PHTF1, SOX5, LCOR	Transcription, nucleus, protein binding, DNA binding, phosphoprotein
Group 3 (13.56)	BUB1B, LATS1, CSNK1A1, RPS6KA3, SGK3, LIMK1, IRAK1, PRKCE, CDK6, TESK2, DYRK3, MAP3K1, WNK3, MAP3K2, WNK1, ROCK2, MAP3K7, CDKL2, CIT, ACVR1C, CDK19, SLK, MAP2K3, SNRK, PIM3, SRPK2, SRPK1, MAP3K13, HIPK3, PDIK1L, MAPK7, MKNK2, NEK1, FRK, CSNK2A1	Kinase activity, transferase, phosphoprotein, binding (protein, ATP, nucleotide), cytoplasm, nucleus, cytosol, protein phosphorylation, proton receptor
Group 4 (11.94)	TGIF1, HOMEZ, ZEB2, SATB1	Transcription activity, nucleus, binding, acetylation, homeobox, phosphoprotein, repressor
Group 5 (11.74)	RAPH1, OSBPL3, PLEKHA8, SKAP2, EXOC8, OSBP, SPATA13	Membrane, transport, cytosol, cytoplasm, protein binding, pleckstrin homology, phosphoprotein
Group 6 (11.44)	RLIM, RNF103, TRIM38, BMI1, RFFL, UBR3, RNF6, TRIM33, TRIM59, RNF185, PCGF6, RNF111, MARCH3, CBLL1, RNF11, DTX3L, RSPRY1, TOPORS, TRIM4, TRIM2, RNF141, SCAF11, MYCBP2	Zinc, zinc finger, binding (metal, protein, ion), ligase activity, phosphoprotein, nucleus, Ubl conjugation pathway, RING
Group 7 (11.15)	NCL, CPEB4, RBM27, MYEF2, MSI2, ELAVL4, RBM39, SREK1, SRSF7, HNRNPH1, CPEB3, CELF2, PTBP3, PPIL4, HNRNPA3, SRSF11, HNRNPR	RNA/nucleotide/nucleic binding, protein binding, nucleus, cytoplasm, acetylation, mRNA processing, nucleoplasm
Group 8 (9.92)	ZMAT5, MBNL1, SNRNP48, SCAF11	Spliceosome, metal/zinc/ion binding, protein binding, nucleus, cytoplasm, RNA binding, RNA splicing, mRNA processing, phosphoprotein, zinc finger
Group 9 (9.74)	ZNF667, RSF1, ZBTB44, ZBTB8A, PHF20L1, WHSC1L1, ZNF662, ZBTB47, REST, ZNF654, ZNF35, ZNF587, ZNF326, ZNF573, ZNF704, ZNF217, ARID2, ZNF429, ZNF24, RREB1, ZNF277, ZNF207, SNAI1, ZNF268, ZNF460, NR2C2, ZNF200, ZEB2, ZUFSP, SUZ12, ZNF292, TSHZ3, THAP1, SNAI2, ZNF646, ZNF607, HIC2, BCL11B, ZNF652, BCL6, ZNF367, PCGF6, KAT7, GLIS3, ATMIN, PHF20, ADNP, KLF5, KLF9, ZNF264, SP1, GLIS2, KAT6A, ZBTB20, ZNF148, ZNF532, TRPS1, TRIM33, BAZ1B, IKZF3, ZNF451, ZNF440, LCOR, ZBTB38, ZEB1	Zinc finger, zinc, nucleus, transcription, binding (meta, protein, ion, DNA, nucleic acid), phosphoprotein
Group 10 (9.29)	USP7, USP47, USP42, USP34	Cytosol, cytoplasm, nucleus, DNA repair, ubiquitin activity, protease, hydrolase, phosphoprotein, acetylation
Group 11 (9.13)	NR2C2, ESR1, PPARA, NR2F2, NR3C1, HNF4A	Regulation of transcription, acetylation, cytoplasm, nucleus, protein/metal/DNA/ion binding, receptor, lipid metabolic process, activator, phosphoroprotein, disease mutation, nucleoplasm
Group 12 (8.74)	SMAD2, SMAD3, SMAD4, SMAD7, SMAD9	Signalling pathway, binding, cell cycle, transcription, nucleus, cytoplasm, cancer, disease mutation
Group 13 (8.55)	RPS4X, RPS7, RPS19, RPS27, RPS2, RPL35A, RPSA, RPL24	Nucleus, cytoplasm, phosphoprotein, extracellular matrix, binding translation, ribonucleoprotein, ribosomal protein, membrane, cytosol, exosome
Group 14 (8.54)	DDX3X, EIF4A2, SMARCA4, DHX33, ATRX, DDX46, DDX55, DDX3Y, CHD9, DDX6	Cytoplasm, nucleus, binding, phosphoprotein, helicase, hydrolase, ubl conjugation
Group 15 (8.26)	TTC38, TTC33, SGTB, FKBP5	Extracellular exosome, chaperone, acetylation, phosphoprotein, tetratricopeptide, protein binding
Group 16 (7.89)	TGIF1, MEIS1, HOXA1, EN2, PBX1, HOXA9, PKNOX1	Transcription, DNA binding, organism development, homeobox, nucleus
Group 17 (7.74)	ANKRD9, CLIP4, RAI14, SOWAHC, HECTD1, ANKRD46, ANKRD13B	Phosphoprotein, Ankyrin
Group 18 (7.74)	SETD2, WHSC1, WHSC1L1, TRIM33, SETD1B	Transcription regulation, zinc finger, chromosome, transferase, nucleus, phosphoprotein, isopeptide bond, Ubl conjugation, associated with SET domain
Group 19 (7.00)	ARHGEF28, FGD6, RALGPS2, NET1, SPATA13, ARHGEF12	Cell membrane, cytosol, cytoplasm, signal transduction, Rho guanyl-nucleotide exchange factor, phosphoprotein
Group 20 (6.74)	DOCK4, RALGPS2, DOCK7, DOCK10, DOCK5	Cytoplasm, cell membrane, acetylation, dedicator of cytokinesis, GTPase activity, phosphoprotein, DOCK-homology region, intracellular
Group 21 (6.48)	KIF13A, KIF5B, KIF1C, KIF2A, DYNC1LI2	Membrane, centrosome, methylation, ATPase activity, kinesin, cytoplasm, ATP binding, cytoskeleton, coiled coil, microtube
Group 22 (6.13)	AKT2, PIK3CA, HRAS, AKT1, MAPK1, PIK3R1, MAPK9, MAPK8	Signalling pathway (GnRH, MAPKinase, EGF, PDGF, IGF-1, insulin, CXCR4, Rrk, PI3, ERBB2, Trka receptor, Ras, Jak-STAT, AMPK, ErbB, cAMP, Toll-line receptor), binding, disease, cancer, cytosol, kinase activity, nucleus, cytoplasm, acetylation, infection, apoptosis,
Group 23 (5.99)	BMPR2, BMPR1B, BMPR1A, TGFBR2, ACVR1C, ACVR2B, DDR2	Phosphoprotein, phosphorylation, kinase activity, signalling pathway (Hippo, BMP, regulation of stem cell, TGF-beta), binding (ATP, metal, nucleotide, ion, protein) TGF-beta receptor, phosphorylation, disease mutation, disulphide bond, transferase, receptor, membrane, transmembrane, signal, extracellular
Group 24 (5.41)	EGFR, PDGFRA, EPHA4, ERBB3, ERBB2, CSF1R, DDR2, IGF1R	Membrane, transmembrane, receptor, signal, binding (ATP, nucleotide, protein), kinase, signalling pathway (Ras, Rap1, calcium, cancer), wound healing, cancer, endocytosis, transferase, microRNAs in cancer, cytoplasm, phosphorylation activity, glycoprotein, extracellular, cytoplasm, disease mutation
Group 25 (5.19)	RASGRP3, RALGPS2, RAPGEF6, RGL2	Cytoplasm, membrane, signalling pathway (Ras, Rap1,) binding (Ras GTPase, protein) signal transduction, GTPase activity, phosphoprotein, Ras guanine nucleotide exchange factor
Group 26 (4.74)	STXBP5, WDR7, WSB1, CORO2A, DCAF10, WDR77, PHIP, BRWD3, GNB4, NBEA, ELP2, DCAF8, FBXW7, TBL1XR1, TAF5	WD (1, 2,3,4,5,6,7, 40), WD repeat, phosphoprotein
Group 27 (4.14)	DUSP5, PTPDC1, DUSP8, DUSP10	Phosphatase activity, cytoplasm nucleus, nucleoplasm, hydrolase, MAPK signalling pathway, Rhodanese, dephosphorylation
Group 28 (3.49)	RHOB, RAB6C, RHOQ, RAB22A, RAB33B, RAB6A, RAB5B, RAP2B, RAB44	Cytosol, membrane, transport, methylation, GTPase activity, extracellular exosome, binding (protein, nucleotide, GTP, phosphate, lipid), lipoprotein, prenylation
Group 29 (3.12)	SOCS5, SOCS4, SOCS3, SOCS6	Protein binding, growth regulation, regulation of Jak-STAT, signal transduction inhibitor, signalling pathway (STAT, prolactin, cytokine-mediated, insulin), cytoplasm, SOCS box, SH2 domain, Ubl conjugation pathway, intracellular, inflammation response, suppressor of cytokine signalling, type II diabetes mellitus, protein ubiquitination
Group 30 (2.77)	LCLAT1, SERINC1, SERAC1, LPGAT1, TMEM147	Membrane, transmembrane, lipid metabolism, lipid biosynthesis, phospholipid metabolism, phospholipid biosynthesis, protein binding, endoplasmic reticulum membrane
Group 31 (2.73)	LRRC20, FBXL2, LRRC57, TBCEL, CEP97, SKP2, FBXL5, VASN, FBXL13, FMOD, GP5, ZYG11B, FBXL3, LRRC1, CNTRL, LRRC2	Leucine-rich repeat (1, 2, 3, 4, 5, 6, 7, 8), protein binding
Group 32 (2.57)	COL4A1, COL3A1, COL5A2, COL5A1, COL1A1	Extracellular matrix, signal, disease mutation, extracellular region, fibrillar collagen, focal adhesion, collagen, secreted, binding (metal, ion, platelet-derived growth factor), PI3-Akt signalling pathway, calcium, glycoprotein, skin development, disulphide bond, glycosylation, skeletal system development
Group 33 (1.50)	IVNS1ABP, KLHL24, KBTBD6, KBTBD7, IPP, KLHL15, KLHL28	Kelch (1,2,3,4,5,6), kelch repeat, kelch-like protein, protein ubiquitination, ubiquitineous activity, BTB domain, BTB/POZ
Group 34 (1.25)	MMP13, PAPPA, MMP10, MMP9, ADAMTS4, MMP2, MMP1	Zinc, calcium, signal, membrane, extracellular matrix, collagen degradation, hemopexin-like domain, metallopeptidase, glycoprotein, zymogen, protease, hydrolase, disulphide bond, metal binding
Group 35 (0.93)	CERS4, SPTLC3, SGPL1, SPTLC2, CERS6	Transmembrane, metabolic pathway, sphingolipid activity, pyridoxal phosphate, endoplasmic reticulum, ceramide biosynthetic process
Group 36 (0.01)	EDNRA, C15orf48, ARMCX3, VOPP1, ANKRD46, TMPPE, SLC2A14, SACM1L, TMEM2, ST6GAL1, FAM20B, SAMD5, SLC31A1, GLRA3, SLC12A5, TMEM178B, BOC, SLC16A10, FGFRL1, TM9SF3, IL13RA1, LIFR, B3GNT5, LMBR1, TMEM170A, IL10RB, PIGX, GALNT6, CNNM3, SLC17A5, DSE, SLC44A1, SGCB, SLC39A9, CERS6, FAXC, FAXDC2, TMEM56, CHST10, TMED4, MCTP1, SLC26A2, TMEM147, B3GALNT1, HTR2C, MRAP2, PGRMC2, BSG, SFXN1, CADM2, SFT2D2, SLC45A4, COX20, PEAR1, TOR1AIP2, CCR7, CCR6, CCR5, CCR1, NCSTN, NETO2, HEPHL1, OR7D2, BTN3A3, GJD2, CYBRD1, PIGN, LRIT3, CLPTM1L, MGAT4A, CDH7, HERPUD2, MXRA7, TMEM245, MEGF9, GPR156, SLMAP, PIGP, HSP40, GXYLT2, TMEM97, SLC5A3, EXT1, MOXD1, SERINC1, SCN2B, CD151, HS3ST3B1, GXYLT1, TNFRSF10B, TMEM120B, ORAI2, OLR1, TNFRSF10D, PTGFR, CD47, CD44, RER1	Membrane, transmembrane, glycoprotein, cytoplasmic domain, glycosylation

**Table 5:** BP terms related to migration.

Terms	*p*-adj	Terms	*p*-adj
**Target Genes Associated with HaCaT Cell-Derived EV miRNAs**			
Regulation of cell migration	0.0033	Epithelium migration	0.0382
Negative regulation of cell migration	0.0301	Regulation of epithelial cell migration	0.0398
Epithelial cell migration	0.0352	Tissue migration	0.0465
**Target Genes Associated with Primary Keratinocyte-Derived EV miRNAs**			
Regulation of cell migration	0.0001	Regulation of mononuclear cell migration	0.0032
Positive regulation of cell migration	0.0001	Regulation of vascular associated smooth muscle cell migration	0.0063
Positive regulation of epithelial cell migration	0.0001	Regulation of blood vessel endothelial cell migration	0.0088
Regulation of epithelial cell migration	0.0001	Regulation of fibroblast migration	0.0092
Positive regulation of endothelial cell migration	0.0001	Regulation of leukocyte migration	0.012
Regulation of endothelial cell migration	0.0001	Positive regulation of vascular associated smooth muscle cell migration	0.0161
Cell migration	0.0001	Leukocyte migration	0.0169
Positive regulation of mononuclear cell migration	0.0002	Thymocyte migration	0.0192
Regulation of smooth muscle cell migration	0.0007	Positive regulation of fibroblast migration	0.0211
Positive regulation of leukocyte migration	0.0011	Regulation of trophoblast cell migration	0.0411
Negative regulation of cell migration	0.0011	Positive regulation of cell migration involved in sprouting angiogenesis	0.0411
Positive regulation of blood vessel endothelial cell migration	0.0017	Dendritic cell migration	0.0423
Positive regulation of smooth muscle cell migration	0.0032		

## Supplementary Materials

The following are available online at https://www.mdpi.com/1422-0067/21/3/1141/s1, Supplemental Table S1: Target genes regulated by hsa-miR-205, Supplemental Table S2: Target genes regulated by hsa-miR-21, by hsa-miR-203, by hsa-miR-21 and by hsa-miR-143, Supplemental Table S3: Biological processes associated with target genes regulated by hsa-miR-205.

## Abbreviations

AP(s)	Apoptotic Bodies
BP	Biological process
CM	Conditioned medium
ECM	Extracellular matrix
EX(s)	Exosome(s)
EV(s)	Extracellular membrane vesicle(s)
GO	Gene Ontology
MAPK	Mitogen-activated protein kinase
miRNA	microRNAs
MV(s)	Microvesicle(s)
PCA	Principal component analysis
PBS	Phosphate -saline
PKC(s)	Primary keratinocyte(s)
PTEN	Phosphatase and tensin homolog
RPM	Read per million
SHIP2	SH2-domain containing inositol 5-phospatase 2
TGF-beta	Transforming growth factor beta
