# Supplementary material for: Deep Sequencing MicroRNAs from Extracellular Membrane Vesicles Revealed the Association of the Vesicle Cargo with Cellular Origin
Source: Int J Mol Sci. 2020 Feb 8;21(3):1141. doi: 10.3390/ijms21031141 (PMC7036882; doi:10.3390/ijms21031141)
Supplement: Supplementary file 1 [file ijms-21-01141-s001.pdf]

Supplemental table 1: Target genes regulated by hsa-miR-205

Index	Target gene	Index	Target gene	Index	Target gene	Index	Target gene	Index	Target gene
1	KCTD20	35	UBE2Z	69	SLC38A1	103	LPCAT1	137	STK38L
2	MAPK14	36	YWHAH	70	ANGPTL7	104	MARCKS	138	C1orf123
3	TXNL1	37	RBBP4	71	CTGF	105	MED13	139	GUCD1
4	SPDL1	38	LRP1	72	CYR61	106	IPO7	140	CDK6
5	TCF20	39	IMPAD1	73	TP73	107	PHC2	141	CDKN2AIPNL
6	RAN	40	GNAS	74	EGLN2	108	PICALM	142	CLIP1
7	RGS6	41	MED1	75	ERBB2	109	PLAGL2	143	CUL5
8	HOXA11	42	INPPL1	76	PRRG4	110	NDUFA4	144	C6orf201
9	PAPPA-AS1	43	DDX5	77	F2RL2	111	NDUFB2	145	VTI1A
10	PRR15	44	E2F1	78	GOT1	112	NIPA2	146	SLC5A12
11	ACTRT3	45	E2F5	79	NUFIP2	113	NOTCH2	147	MAML2
12	YES1	46	ZEB2	80	IL24	114	PANK1	148	MAP3K9
13	SRC	47	ERBB3	81	IL32	115	PARD6B	149	NUDT21
14	NPRL3	48	PRKCE	82	RNF217	116	TMEM66	150	DNAJA1
15	NFAT5	49	SLC41A1	83	ZNF585B	117	EZR	151	CCDC108
16	XPOT	50	SLC7A2	84	SIGMAR1	118	ENPP4	152	SHISA6
17	KCTD16	51	ZEB1	85	VEGFA	119	LRRTM4	153	ACP1
18	TMSB4X	52	PHF8	86	BCL9L	120	KCNJ10	154	BCL2
19	PLCXD2	53	TMEM201	87	CREB1	121	PHLPP2	155	NCAPG
20	TNFSF8	54	PTPRJ	88	SERINC3	122	YEATS2	156	KLHL5
21	SLC25A25	55	ETNK1	89	HMGB3	123	VAMP1	157	ACSL4
22	C11orf74	56	XPR1	90	SRD5A1	124	RTN3	158	BCL6
23	GM2A	57	MRPL44	91	PTEN	125	RFX7	159	ITGA5
24	SMNDC1	58	TM9SF2	92	ESRRG	126	RAP2B	160	ACSL1
25	BAMBI	59	PAIP2B	93	PRLR	127	TRAF3IP1	161	EID2B
26	LCOR	60	NEK9	94	ICK	128	SERTAD2	162	TEX35
27	TMEM239	61	NOX5	95	LOH12CR1	129	TOLLIP	163	YY1
28	AMOT	62	DMXL2	96	SLC39A14	130	TMEM55B	164	SMAD1
29	CDK1	63	ETF1	97	BDP1	131	TMEM123	165	SMAD4
30	SQLE	64	LAMC1	98	MMD	132	TAF11	166	PTPRM
31	CPEB3	65	LRRK2	99	MGLL	133	AFF4	167	AR
32	VPS52	66	SMIM13	100	LYN	134	AFF1	168	PHF12
33	JMJD1C	67	DHCR24	101	LYSMD3	135	B4GALT5		
34	NSF	68	RAB11FIP1	102	LRRC59	136	B4GALT6		

Supplemental table 2: Target genes regulated by hsa-miR-21, by hsa-miR-203, by hsa-miR-21 and by hsa-miR-143

Index	Target gene	Index	Target gene	Index	Target gene	Index	Target gene	Index	Target gene
1	DNAAF2	217	IPMK	433	HAAO	649	ZBTB38	865	EDC3
2	CXXC4	218	RASA1	434	DCAF16	650	SLMAP	866	NR3C1
3	CIT	219	HDAC4	435	KIF5B	651	APC	867	TSC22D4
4	DDX55	220	COX2	436	VSNL1	652	SAR1A	868	SOGA1
5	GLRA3	221	BMPR2	437	GLUL	653	PIGX	869	PDHA1
6	CASK	222	SZRD1	438	C10orf137	654	SMC1A	870	E2F2
7	OSBP	223	UFL1	439	PLEKHA8	655	BDH2	871	SRSF7
8	CDKN1A	224	FAM208A	440	PPARA	656	NETO2	872	ZNF217
9	SRC	225	BMPR1A	441	ESR1	657	ALMS1	873	LEMD3
10	TET2	226	XIAP	442	FGFRL1	658	TOR1AIP2	874	CCNT2
11	CD44	227	CALM3	443	SLC16A10	659	B3GNT5	875	FOXP1
12	BRAF	228	LOH12CR2	444	APAF1	660	GLG1	876	ARID5B
13	TCF4	229	RGL2	445	GLCCI1	661	CEP152	877	TBX3

14	NFYA	230	FOXO3	446	RP2	662	HMGB3	878	MIS18BP1
15	MAP3K13	231	RER1	447	SGK3	663	SLK	879	CSNK2A1
16	RASAL2	232	MSH6	448	FAS	664	FILIP1L	880	MAX
17	ZEB1	233	MSH2	449	FAM3C	665	DCP1A	881	PRKACA
18	SMAD9	234	LCE1A	450	HIPK3	666	WHSC1L1	882	BTF3
19	BCL11B	235	RNF41	451	BTG2	667	VASH2	883	TBC1D12
20	FOXK1	236	JMY	452	SOCS5	668	WNK3	884	ZFYVE20
21	PRKCA	237	HNRNPK	453	SESN1	669	MEGF9	885	LATS1
22	ASAP1	238	TOPORS	454	FBXL3	670	TNPO1	886	CD47
23	HPGD	239	DAXX	455	SMCHD1	671	FERMT2	887	CPEB3
24	CAV1	240	TGFBR3	456	ATXN10	672	KAT6A	888	RMND5A
25	RREB1	241	TP63	457	SEPT2	673	TNFRSF11B	889	KBTBD7
26	SMN1	242	TP53BP2	458	IGFBP5	674	WWC2	890	MEIS1
27	CD151	243	PPIF	459	FNDC3B	675	PTPDC1	891	GNB4
28	RAN	244	BCL2L2	460	PDCD4	676	CCNG1	892	MMP2
29	WNT1	245	MYCBP	461	ABL1	677	SCRN1	893	TXLNG2P
30	COL5A1	246	TIAM1	462	PARK7	678	CDC25A	894	REV3L
31	MAPK1	247	SCD	463	MAPK7	679	BCL2	895	VEGFA
32	CHST10	248	EYA4	464	SERPINB5	680	GALNT6	896	PARP1
33	MAP3K7	249	EDNRA	465	RHOB	681	PPM1L	897	CERS6
34	PDGFRA	250	GDAP1	466	RPS19	682	MMP9	898	SCAF11
35	SMAD3	251	MEF2C	467	ANP32A	683	SMNDC1	899	HNRNPH1
36	CTNND1	252	ISCU	468	GPCPD1	684	NCSTN	900	TGFB1
37	SIX1	253	PPM1D	469	RBL1	685	CAPRIN1	901	SNRNP48
38	ZNF646	254	HRAS	470	SRPK1	686	CNTRL	902	FUBP1
39	ZNF24	255	IGF1R	471	ZCCHC3	687	PARP9	903	SOX2
40	PLD2	256	ACVR1C	472	ZNF277	688	CKAP5	904	GTF2A1
41	CYR61	257	EIF4A2	473	IFT140	689	PHTF1	905	RRAGC
42	DUSP5	258	FSCN1	474	MIER3	690	MRAP2	906	RAPH1
43	OR7D2	259	ANKRD46	475	UVRAG	691	MYCBP2	907	CYBRD1
44	PDE7A	260	EGFR	476	TIMP3	692	ITSN2	908	SLAIN2
45	CASP5	261	RGS2	477	MTAP	693	BCAT1	909	KIAA1551
46	FBXL13	262	MAOA	478	SOX5	694	PPFIA4	910	PIGN
47	IRAK1	263	HTR2C	479	RECK	695	YME1L1	911	HPS5
48	VHL	264	BDNF	480	SLC30A9	696	ZNF667	912	SESTD1
49	GDF5	265	ZEB2	481	FMOD	697	ETNK1	913	NBEA
50	HNRNPA3	266	BMI1	482	TGFBR2	698	CYP4V2	914	TAF5
51	FBXL5	267	SMAD4	483	E2F1	699	BRCA1	915	ENAH
52	HOXA1	268	RUNX2	484	PTEN	700	DOCK10	916	TBL1XR1
53	WWC1	269	MMP10	485	SON	701	SACM1L	917	ELOVL4
54	LRIT3	270	DLX5	486	TGFBI	702	ATAD2B	918	EPHA4
55	MIDN	271	IL6	487	MARCKS	703	CALD1	919	MEF2A
56	GREM1	272	MMP1	488	LRRFIP1	704	LYRM7	920	ZBTB47
57	TUBB2A	273	IL1B	489	FOXK2	705	TRAPPC2	921	AP3M1
58	TRIML2	274	ICAM1	490	FOXN2	706	LIFR	922	GNE
59	JUN	275	PLAT	491	FBXW7	707	RAB6C	923	USP34
60	LASP1	276	PTX3	492	LCLAT1	708	TSNAX	924	CLOCK
61	HDAC6	277	TNFAIP3	493	CDC42SE2	709	FIGN	925	ZBTB8A
62	RAB44	278	CCR1	494	C15ORF48	710	EPM2A	926	DDR2
63	NCOA4	279	CDK2AP1	495	NFIB	711	PIK3R1	927	TRIM38
64	CDKL2	280	NCOA1	496	C8orf17	712	RPS6KA3	928	KIAA1715
65	NAPEPLD	281	CMPK1	497	BMP7	713	ADNP	929	VPS54
66	ARHGEF28	282	TRIM4	498	TPM1	714	GXYLT2	930	LIN7C
67	CERKL	283	PURG	499	PPP2R5E	715	PBRM1	931	BAZ1B
68	POLR1B	284	SPATA18	500	RRP7A	716	LRRC57	932	MGAT4A
69	YWHAZ	285	PIGP	501	VGLL4	717	DUSP8	933	IREB2
70	WWP1	286	GSTO2	502	FAM136A	718	KLHL15	934	DTX3L
71	SATB1	287	STX4	503	PIP5K1A	719	MPP5	935	DDHD2

72	SDC1	288	RPSA	504	PI4K2B	720	TAF1	936	NIPBL
73	SLC12A5	289	OTUD1	505	ZNF264	721	B3GALNT1	937	LPGAT1
74	GINS2	290	TMEM147	506	GK5	722	VPS13A	938	EDIL3
75	SRGAP1	291	RBM39	507	SH3BP4	723	GPD1L	939	FANCI
76	LRRC20	292	NUP214	508	KIAA0408	724	SLC5A3	940	SGCB
77	FRK	293	HSPA1B	509	MXRA7	725	PRKCE	941	C2orf43
78	GNAS	294	ELP5	510	HIC2	726	STRBP	942	LRRC1
79	KLK2	295	GLIS2	511	GLRX2	727	SEC63	943	H3F3B
80	ADH5	296	DNHD1	512	FLYWCH2	728	TOP2A	944	RAB5B
81	CCR5	297	PEX5	513	ADAMTS4	729	HECTD1	945	FRAT2
82	PDIK1L	298	VAPB	514	DBT	730	SSFA2	946	C5orf24
83	CCSAP	299	RPL35A	515	SAMD8	731	GPD2	947	PPAP2A
84	ARID1A	300	BUB1B	516	EXOC2	732	EIF5	948	NIN
85	TMED4	301	RPL24	517	RNF141	733	HAPLN1	949	TPRG1L
86	DLC1	302	RPS4X	518	SLC45A4	734	SFXN1	950	COL5A2
87	ABAT	303	RPS2	519	GXYLT1	735	PAG1	951	C20orf194
88	BRWD3	304	KCTD12	520	ZWINT	736	PTK2	952	DAAM1
89	CPEB4	305	LBP	521	SCN2B	737	DYNC1LI2	953	BOC
90	CTC1	306	CLPTM1L	522	ZC3HAV1L	738	NEK1	954	PALLD
91	FRS2	307	PRRC2B	523	KCTD10	739	ACTR2	955	PHIP
92	SUMO1	308	MCM9	524	NUP50	740	CSNK1A1	956	KLHL24
93	RAPGEF1	309	PARD6B	525	BOLA3	741	APOLD1	957	MOAP1
94	MAPK8	310	KIF1C	526	KCNJ2	742	PTBP3	958	WHSC1
95	GP5	311	PAPPA	527	SPATA13	743	UBR3	959	ZNF326
96	GGA2	312	CYP20A1	528	C15orf52	744	CCT6P1	960	BTBD7
97	LCOR	313	SPTLC2	529	TFPI	745	TMX4	961	ACAT1
98	NUDT3	314	GINS4	530	SYNM	746	DMD	962	PFKFB2
99	DAZAP2	315	HELZ	531	TMEM178B	747	ZFYVE16	963	PDGFD
100	PLEKHM3	316	TSC22D2	532	TIMM8A	748	FNBP1	964	COBLL1
101	HNRPDL	317	ZMAT5	533	FMNL2	749	ZBTB20	965	SAMD5
102	PLEKHA2	318	SPRY4	534	ARID3B	750	FAM217B	966	EXOC8
103	TBCEL	319	PTGS2	535	KLK10	751	DDX3X	967	RB1
104	BCL7A	320	TJP2	536	HTR2A	752	ZADH2	968	ASRGL1
105	THRAP3	321	LINC00598	537	CAMK2N1	753	TMEM56	969	SRSF11
106	DNAJC16	322	TCEAL1	538	BTG1	754	SPTLC3	970	UTRN
107	DICER1	323	CREB1	539	ZBTB44	755	TRIM59	971	IVNS1ABP
108	OAS3	324	SMARCA4	540	SLC39A9	756	MTMR12	972	OSBPL1A
109	CENPQ	325	PTMS	541	BTF3L4	757	KBTBD6	973	RSPRY1
110	CCL1	326	MACC1	542	ZNF268	758	TNRC6B	974	ANKRD28
111	CCR7	327	NFKB1	543	PITHD1	759	SLC9A6	975	SYNE2
112	BNIP2	328	SLC23A1	544	SYK	760	ZNF207	976	DDX3Y
113	AP1AR	329	PCGF6	545	ZBED3	761	RALGPS2	977	PTAR1
114	SET	330	ZNF148	546	PGLS	762	FAM46A	978	PAN3
115	SGTB	331	BIRC5	547	ZNF654	763	NFAT5	979	MYEF2
116	SPPL3	332	PIAS3	548	TMEM70	764	KLHDC5	980	PKD2
117	RNF103	333	ZNF704	549	SMURF2	765	SKP2	981	RASEF
118	RNF111	334	HK2	550	SH3GLB1	766	GPR64	982	ZRANB1
119	NSUN2	335	SERPINE1	551	CNNM4	767	KLF5	983	MON2
120	ENO4	336	DOCK7	552	MSI2	768	ST6GAL1	984	TNS3
121	NKX2-1	337	DOCK5	553	OIT3	769	PURB	985	ESYT2
122	SNAI1	338	DOCK4	554	GAS1	770	LCORL	986	GAPVD1
123	RHO	339	DUSP10	555	FYCO1	771	RAB11FIP2	987	RASGRP3
124	C1orf50	340	FHIT	556	ACOT9	772	PKNOX1	988	PHF17
125	HNF4A	341	ZNF573	557	MARCH3	773	ABCD3	989	USP7
126	DCTN6	342	ANKRD9	558	PRAMEF8	774	E2F3	990	GTF2I
127	TPD52L2	343	ZUFSP	559	PRAMEF7	775	ECI2	991	MKNK2
128	C1orf61	344	ZNF607	560	SKAP2	776	ELAVL4	992	CLCN5
129	ZNF440	345	ARPC5	561	HYPK	777	HS3ST3B1	993	GNAQ

130	GAS5	346	CCL20	562	PPIL4	778	GRPEL2	994	HERPUD2
131	TRMT5	347	ERBB3	563	PRKACB	779	NUFIP2	995	MAP3K2
132	TRPS1	348	DGAT2	564	INSIG1	780	AUTS2	996	THOC2
133	C2orf18	349	SOD3	565	EIF1AX	781	TTC33	997	MUC1
134	THAP1	350	FASLG	566	CERS4	782	CORO2A	998	CDK19
135	RUNDC1	351	PRR14L	567	COX20	783	AKAP9	999	ARHGAP21
136	TSR1	352	NTF3	568	HNRNPR	784	NKTR	1000	MDM4
137	RPS27	353	COL4A1	569	LDHA	785	PTPN14	1001	LARS
138	EPM2AIP1	354	IL24	570	GJD2	786	STXBP5	1002	FAXDC2
139	NPPC	355	SOCS6	571	CELF2	787	MGA	1003	TUBGCP5
140	KIAA0930	356	TNF	572	RLIM	788	SPG11	1004	RHOQ
141	ITGB8	357	TAF1D	573	PAX6	789	KIFAP3	1005	TET1
142	IKZF3	358	TMEM120B	574	COMMD2	790	PHF20	1006	CASC5
143	KRIT1	359	ZNF200	575	FGFR1OP	791	CEP97	1007	MOXD1
144	MED9	360	SERBP1	576	PPP1CB	792	DCAF10	1008	LAMP2
145	ZNF429	361	VOPP1	577	NR2F2	793	SRPK2	1009	MRPS10
146	NAA50	362	STOM	578	PNPO	794	TSHZ3	1010	FBXL17
147	LRRC2	363	PEAR1	579	BBC3	795	AIM1	1011	RAI14
148	SLC25A16	364	RASA2	580	IER5	796	DSE	1012	MYO9A
149	GTF2H5	365	FAM71F2	581	KIF13A	797	ZNF292	1013	TMEM2
150	UBE2V2	366	IL7	582	MMP13	798	ZNF587	1014	IPP
151	SLC25A33	367	CUL3	583	ANKRD13B	799	PER3	1015	AHSA2
152	PHAX	368	TPD52L1	584	MAFK	800	SERAC1	1016	PIK3C2A
153	ORC4	369	DYNLT1	585	EN2	801	RUFY3	1017	AGGF1
154	TMPPE	370	ZCCHC11	586	USP42	802	WNK1	1018	PRKAB2
155	TNFRSF10D	371	ASPA	587	NCKAP1	803	NUBPL	1019	SLC26A2
156	NARS	372	ERP44	588	NFIC	804	LIMCH1	1020	SREK1
157	PSAT1	373	NUDCD1	589	DSCR3	805	MTMR9	1021	FAM20B
158	FGD6	374	TRNT1	590	CREG2	806	SEMA5A	1022	ARMCX3
159	CCR6	375	TMEM97	591	CALR	807	PHACTR2	1023	SERPINI1
160	FSD2	376	RPRD2	592	CAND1	808	RABGAP1	1024	DDAH1
161	ORAI2	377	WDR77	593	CBX4	809	VPS36	1025	PROSER1
162	IPO7	378	DDIT4	594	C2orf15	810	FBXL2	1026	FKBP5
163	NET1	379	ACVR2B	595	PIM3	811	WNT5A	1027	NAA30
164	BMPR1B	380	C15orf40	596	ARID2	812	REV1	1028	JPH1
165	BMP6	381	CNNM3	597	C1orf87	813	HOXA9	1029	PRICKLE2
166	SIRT2	382	PRELID2	598	CDH7	814	OSBPL3	1030	TRIM33
167	LIMK1	383	DNAJB6	599	C6orf223	815	APPL1	1031	RAPGEF6
168	PATE2	384	ZNF451	600	G6PC	816	PBX1	1032	TGFB2
169	C20orf24	385	GPR156	601	RBM27	817	ARHGEF12	1033	ZMYM2
170	DDX6	386	MDM2	602	LRAT	818	USP47	1034	MTPN
171	COL3A1	387	SNAI2	603	LONP2	819	BTBD3	1035	GOLGA4
172	TNFRSF10B	388	RCOR1	604	TRIM71	820	ARID4A	1036	EIF4EBP2
173	HOMEZ	389	SLC44A1	605	KLHL28	821	MIB1	1037	LONRF2
174	CLU	390	SP1	606	SCO1	822	FMR1	1038	ELOVL7
175	CSF1R	391	MYD88	607	DYRK3	823	SNX30	1039	PHF16
176	DYT10	392	SMAD7	608	SLC2A1	824	SLC17A5	1040	TRIM2
177	YWHAB	393	CBLL1	609	SMAD2	825	PREPL	1041	SASH1
178	BSG	394	MRO	610	DHX33	826	CCDC14	1042	RAB22A
179	KIF2A	395	FAM120AOS	611	NCL	827	RSF1	1043	PGRMC2
180	CXCL10	396	SOD2	612	BTN3A3	828	SOWAHC	1044	CCDC34
181	IL13RA1	397	FAXC	613	BCL6	829	PLD1	1045	ATP2B4
182	SETD2	398	SLC25A25	614	PDCD10	830	SNRK	1046	POLR3B
183	RNF185	399	PRNP	615	RCC2	831	ATP11B	1047	SPIN1
184	SERINC1	400	SLC2A14	616	RAP2B	832	DLG1	1048	SUZ12
185	NFIA	401	MYPN	617	SEMA3D	833	SLC31A1	1049	GID4
186	RFFL	402	SGPL1	618	NFYB	834	VPS26A	1050	TRPM7
187	ALOX15	403	UQCRB	619	SFT2D2	835	TLR4	1051	PLEKHA1

188	TCEANC2	404	GLIS3	620	RAB33B	836	ZYG11B	1052	MALT1
189	KAT7	405	GABRB1	621	CENPP	837	ATMIN	1053	TMEM245
190	C1orf147	406	MAPK9	622	VASN	838	KLF9	1054	ZNF367
191	UCK2	407	NCALD	623	TTC38	839	OLR1	1055	MORC3
192	NPR1	408	HMGB1	624	ZNF460	840	PURA	1056	MBNL1
193	IFIT1	409	STOX2	625	DNMT3A	841	RAB6A	1057	ZNF35
194	LGALS1	410	RGS17	626	SPRY2	842	MAP3K1	1058	GPAM
195	HIF1A	411	AKT1	627	REST	843	TESK2	1059	FBXO3
196	PRR23A	412	IRF5	628	JAG1	844	EIF2C4	1060	NR2C2
197	IL12A	413	DNTTIP2	629	BASP1	845	PRPF39	1061	FOXN3
198	PIK3CA	414	ROCK2	630	DERL1	846	STAG2	1062	DDX46
199	FGF12	415	TCF21	631	NCAPG	847	ACBD5	1063	DMTF1
200	SETD1B	416	SERTAD3	632	PDHA2	848	UGGT1	1064	CLIP4
201	CYCS	417	ELP2	633	PLOD3	849	AKT2	1065	PM20D2
202	COL1A1	418	RNF6	634	RPS7	850	NT5C2	1066	LMBR1
203	HEPHL1	419	HEXIM1	635	RTN4	851	PRRC1	1067	ATRX
204	CADM2	420	CHD9	636	TM9SF3	852	ZNF532	1068	EXT1
205	TFRC	421	SNX4	637	WFS1	853	AGO2	1069	YARS
206	MYC	422	WSB1	638	WIBG	854	AFTPH	1070	CSRNP2
207	NCOA3	423	MAP2K3	639	KRAS	855	EXOC5	1071	ARPP19
208	TGIF1	424	PER2	640	MYO6	856	ATF2	1072	EFR3B
209	ERBB2	425	ZNF652	641	YOD1	857	PTPN3	1073	ZNF662
210	SOCS3	426	SYT7	642	STAT3	858	PHF20L1	1074	GATA6
211	EIF2S1	427	IL10RB	643	RNF11	859	PTGFR	1075	TRAF3IP1
212	SPATS2L	428	ATF7IP	644	PELI1	860	FAM126B	1076	RASGRP1
213	PCBP1	429	ATM	645	RDH11	861	WDR7		
214	ABCE1	430	MCTP1	646	OSR1	862	DCAF8		
215	SECISBP2L	431	TMEM170A	647	FBXO11	863	UBR5		
216	EGLN1	432	UBXN2A	648	SOCS4	864	CDK6		

Supplemental table 3: Biological processes associated with target genes regulated by hsa-miR-205

**HaCaT**		**Primary keratinocytes**	
Terms	p_adj	*Terms*	p_adj
Positive regulation of macromolecule metabolic process	1.25E-05	Regulation of macromolecule metabolic process	1.32E-29
Response to endogenous stimulus	1.25E-05	Regulation of metabolic process	1.00E-28
Regulation of signaling	1.25E-05	Negative regulation of cellular process	1.21E-28
Positive regulation of cellular metabolic process	1.25E-05	Regulation of cellular metabolic process	8.95E-28
Regulation of cell communication	1.25E-05	Negative regulation of biological process	8.95E-28
Intracellular steroid hormone receptor signaling pathway	1.25E-05	Regulation of primary metabolic process	1.52E-27
Positive regulation of metabolic process	1.39E-05	Regulation of apoptotic process	4.31E-27
Hormone-mediated signaling pathway	1.57E-05	Regulation of programmed cell death	4.78E-27
Cellular response to lipid	1.64E-05	Regulation of gene expression	4.97E-27
Cellular response to endogenous stimulus	7.52E-05	Regulation of cell death	4.17E-26
Positive regulation of phosphorylation	7.53E-05	Regulation of nitrogen compound metabolic process	7.38E-25
Steroid hormone mediated signaling pathway	7.53E-05	Positive regulation of metabolic process	1.75E-24
Response to lipid	8.08E-05	Positive regulation of macromolecule metabolic process	2.40E-24
Positive regulation of gene expression	8.82E-05	Positive regulation of cellular process	5.48E-24
Negative regulation of cell proliferation	8.82E-05	Negative regulation of metabolic process	5.86E-24

Positive regulation of protein phosphorylation	9.09E-05	Positive regulation of cellular metabolic process	5.86E-24
Regulation of signal transduction	9.09E-05	Negative regulation of cellular metabolic process	5.86E-24
Intracellular receptor signaling pathway	9.22E-05	Regulation of nucleobase-containing compound metabolic process	1.68E-23
Positive regulation of RNA metabolic process	9.64E-05	Negative regulation of macromolecule metabolic process	1.71E-23
Positive regulation of protein metabolic process	1.21E-04	Negative regulation of apoptotic process	4.45E-23
